# An Apoplastic Defensin of Wheat Elicits the Production of Extracellular Polysaccharides in Snow Mold

**DOI:** 10.3390/plants10081607

**Published:** 2021-08-05

**Authors:** Ayako Isobe, Chikako Kuwabara, Michiya Koike, Keita Sutoh, Kentaro Sasaki, Ryozo Imai

**Affiliations:** 1 Hokkaido Agricultural Research Center, National Agriculture and Food Research Organization (NARO), Toyohira-ku, Sapporo 062-8555, Japan; isoaya@affrc.go.jp (A.I.); ck-ukz@for.agr.hokudai.ac.jp (C.K.); koike-michiya@hokuren.jp (M.K.); sutoh@life-science.co.jp (K.S.); skentaro@affrc.go.jp (K.S.); 2 Graduate School of Life Science, Hokkaido University, Kita-ku, Sapporo 060-0810, Japan; 3 Institute of Agrobiological Sciences, National Agriculture and Food Research Organization (NARO), 3-1-3 Kannondai, Tsukuba 305-8604, Japan

**Keywords:** apoplast, cold acclimation, defensin, extracellular polysaccharides, wheat

## Abstract

TAD1 (*Triticum aestivum* defensin 1) is a plant defensin specifically induced by low temperature in winter wheat. In this study, we demonstrated that TAD1 accumulated in the apoplast during cold acclimation and displayed antifungal activity against the pink snow mold fungi *Microdochium nivale*. When *M. nivale* was treated with TAD1, Congo red-stainable extracellular polysaccharides (EPS) were produced. The EPS were degradable by cellulase treatment, suggesting the involvement of β-1,4 glucans. Interestingly, when the fungus was treated with FITC-labeled TAD1, fluorescent signals were observed within the EPS layer. Taken together, these results support the hypothesis that the EPS plays a role as a physical barrier against antimicrobial proteins secreted by plants. We anticipate that the findings from our study will have broad impact and will increase our understanding of plant–snow mold interactions under snow.

## 1. Introduction

Overwintering cereals and perennial grasses in temperate and boreal climate zones must endure prolonged exposure to subzero temperatures. The freezing tolerance of plants substantially increases after a period of exposure to low but non-freezing temperatures, which is a well characterized process known as cold acclimation [1]. In northern areas with deep and persistent snow, winter cereals such as wheat and rye often suffer from snow mold diseases caused by fungi such as *Microdochium nivale*, *Typhula ishikariensis*, and *Sclerotinia borealis*, while they are protected from freezing under the snow cover. It has been reported that resistance against snow mold fungus increases during cold acclimation in overwintering plants such as winter wheat [2], barley [3], and Arabidopsis [4]. However, the mechanism of cold-induced resistance to pathogens is poorly understood.

In order to better understand this process and elucidate the functional mechanisms of this phenomenon, we aimed to clone and functionally characterize novel defense-related genes that are induced during cold acclimation. We identified a plant defensin gene, *TAD1* (*Triticum aestivum* defensin 1), that is specifically induced by cold treatment in winter wheat [5]. Plant defensins are known as a class of the pathogenesis-related (PR) proteins [6]. Under in vitro conditions, the recombinant TAD1 protein showed inhibitory activity against the plant pathogen *Pseudomonas cichorii* and the snow mold *T. ishikariensis* [5,7]. Furthermore, overexpression of *TAD1* confers resistance against *T. ishikariensis* in wheat [7]. *TAD1* is induced by cold and abscisic acid treatment but not by defense-related phytohormones such as salicylic acid and methyl jasmonate, suggesting that the gene is under regulation of abiotic signals rather than biotic ones [5].

Here, we report that the TAD1 protein accumulates in apoplasts of wheat leaf tissue during cold acclimation and suggest its involvement in cold-induced resistance against snow molds. We found that *M. nivale* secretes extracellular polysaccharides in response to TAD1 treatment, providing functional evidence for the existence of a defense mechanism of the pathogen against host defense systems.

## 2. Results

### 2.1. TAD1 Localized in Extracellular Space during Cold Acclimation

**Figure 1:**
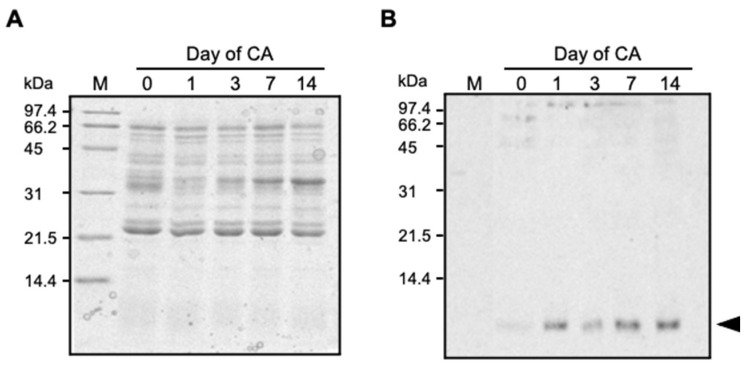
Western blot analysis of cold-acclimated wheat apoplastic protein extracts. (**A**,**B**) Apoplastic proteins from a time course of cold treatment were separated by SDS-PAGE (**A**), and a duplicated gel was transferred to a membrane for Western blot analysis (**B**). CA, cold acclimation.

Since TAD1 has putative N-terminal signal peptides [5], it was expected that TAD1 enters into the secretary pathway. We analyzed the subcellular localization and accumulation of TAD1 during cold acclimation. An immunoblot analysis with polyclonal antibodies against TAD1 did not detect any cross-reacting bands in the total soluble fraction (Figure S1). In contrast, a cross-reacting band with a molecular mass of approximately 6 kDa was detected in the apoplast fractions (Figure 1), which is close in size to that of mature TAD1 protein (5.54 kDa). Accumulation of the TAD1 protein in the apoplast fraction was detectable at the first day of cold acclimation and further accumulated during 14 d of cold acclimation (Figure 1B). This pattern of accumulation was in good accordance with that of *TAD1* expression during cold acclimation [5].

### 2.2. Subcellular Localization of TAD1-GFP Protein

**Figure 2:**
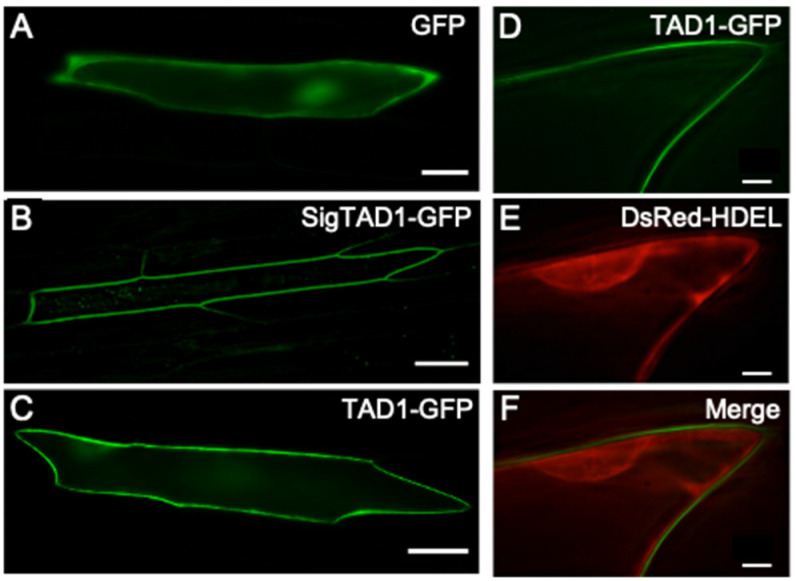
Subcellular localization of GFP fusion proteins in onion epidermal cells. Fluorescent images of the cells transformed with GFP vector (**A**), SigTAD1-GFP (**B**), and TAD1-GFP (**C**). Scale bar for A = 20 µm; scale bar for B and C = 50 µm. (**D**,**E**) Apoplastic localization of the TAD1 protein in onion epidermal cells. Fluorescent images of the cells co-bombarded with TAD1-GFP (**D**) and an ER marker DsRed-HDEL (**E**). (**F**) A merged image of D and E. Scale bar = 10 µm.

The subcellular localization of TAD1 was also analyzed using GFP-fusion proteins. Two expression vectors containing either an N-terminal signal sequence (SigTAD1) or a full length sequence of TAD1 fused with synthetic green fluorescent protein (GFP) (35S::SigTAD1-GFP or 35S::TAD1-GFP) were introduced to and transiently expressed in onion epidermal cells. Localization of the proteins was observed with fluorescent microscopy. When the onion cells were bombarded with the 35S::GFP vector as a control, green fluorescence was observed in the cytoplasm and nucleus (Figure 2A). Onion cells that were bombarded with either 35S::TAD1-GFP or 35S::SigTAD1-GFP displayed GFP fluorescence, which localized in the outer layer of the cell (Figure 2B,C). Since there was little difference between TAD1-GFP and SigTAD1-GFP, it was concluded that the subcellular localization of TAD1 is determined by its N-terminal signal sequence.

To address the TAD1 localization more in detail, we co-expressed an ER marker, DsRed-HDEL, with TAD1-GFP (Figure 2D,E). DsRed-HDEL showed red fluorescence in a typical ER network, including nuclear and plasma membranes (Figure 2E). A merged image clearly showed that TAD1-GFP fluorescence was detected in the outer layer of the cells, suggesting that the TAD1-GFP protein localizes to the cell wall and apoplast (Figure 2F). 

### 2.3. TAD1 Induced Production of Extracellular Polysaccharides in M. nivale

**Figure 3:**
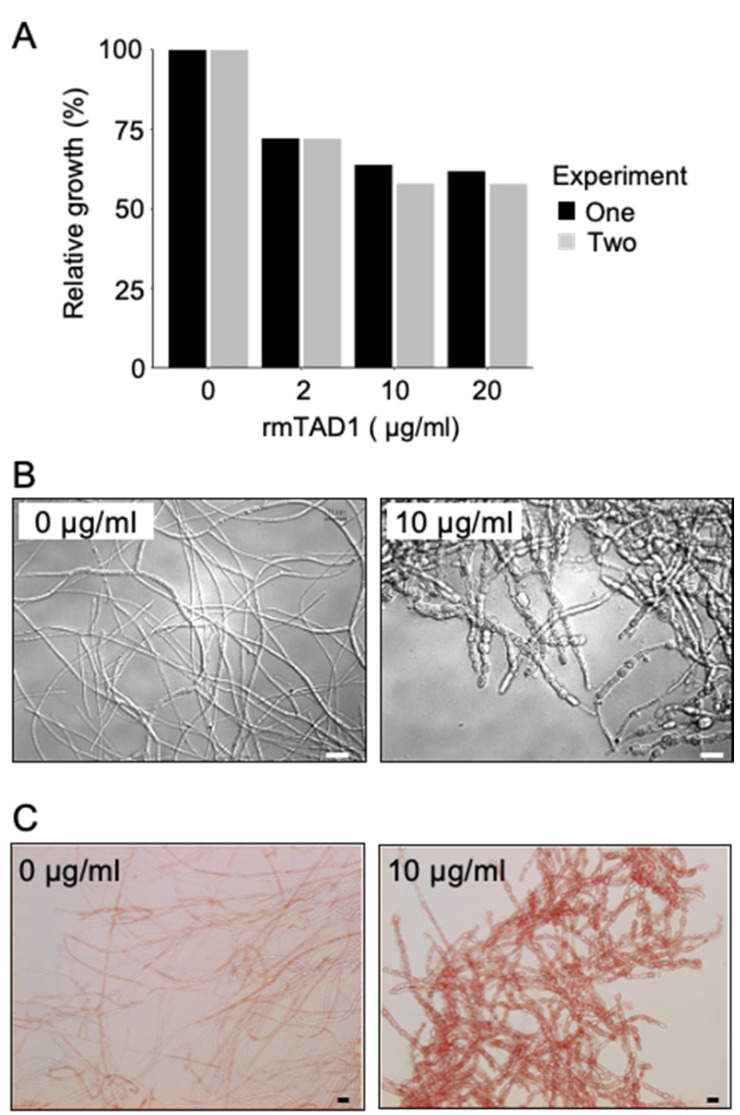
Antifungal activity of TAD1 to snow mold fungus *M. nivale*. (**A**) Relative fungus growth was estimated by cultivation of the *M. nivale* hyphae on a 96-well microplate and increasing the concentration of rmTAD1 (0, 2, 10, and 20 µg/mL) at 10 °C for 9 days. The hyphal growth was monitored by measuring absorbance at 595 nm and was presented as a percentage of the growth in the absence of rmTAD1. Two biological replicate experiments were performed (experiment one and two). (**B**,**C**) Microscopic observation of *M. nivale* hyphae after incubating spores at 10 °C for 9 days with or without rmTAD1 (10 µg/mL). Images of hyphae were captured by using differential interference contrast (DIC) microscopy (**B**). Mycelia were stained with Congo red (**C**). Scale bar = 20 µm.

Antifungal activities of the recombinant mature TAD1 (rmTAD1) against the pink snow mold, *M. nivale*, were determined by hyphal growth inhibition. As shown in Figure 3A, growth of *M. nivale* was inhibited by the addition of rmTAD1, indicating that TAD1 has an antifungal activity against *M. nivale*. When rmTAD1 was added to the culture of *M. nivale*, morphological changes of *M. nivale* were observed, including hyphal aggregation and hyphal tip swelling. In contrast, however, normal hyphal growth was observed without the addition of the protein (Figure 3B). These changes caused by rmTAD1 suggested that TAD1 has a function to make fungus cell division anomalously. More surprisingly, treatment with rmTAD1 induced the accumulation of extracellular substances around hyphae. Subsequent staining with Congo red suggested that the substances contain polysaccharides (Figure 3C). Since it has been previously reported that *M. nivale* uniquely produces extracellular cellulose [8], it is plausible that the produced extracellular polysaccharides (EPS) are cellulose. These results suggested that *M. nivale* produces EPS in response to a plant defense protein TAD1.

**Figure 4:**
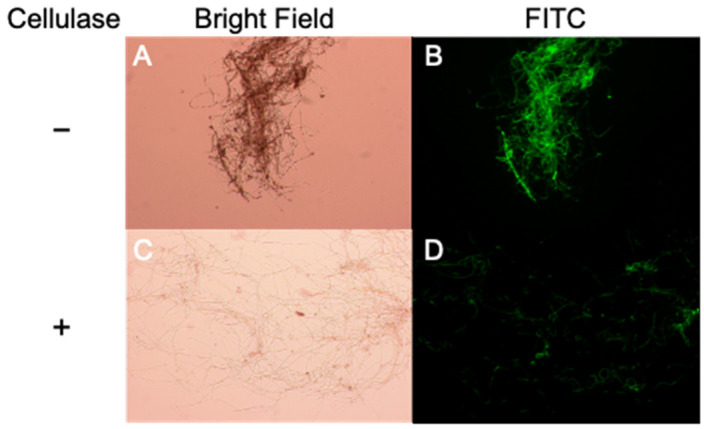
TAD1 accumulated to a high level within the EPS layer. Microscopic observation of *M. nivale* mycelia after incubating spores at 15 °C for 7 days with FITC-labelled rmTAD1 (10 µg/mL) through bright field and FITC filter sets. The first row (**A**,**B**) represents the images without cellulase. The second row (**C**,**D**) represents the images with cellulase.

In some bacteria, EPS are involved in the protection against chemical and biological attacks [9,10]. Consequently, we therefore tested the possibility that EPS produced by *M. nivale* has a protective role against the plant defense system. To address the interaction between TAD1 and EPS, we labeled rmTAD1 with fluorescein isothiocyanate (FITC). Functionality of the rmTAD1-FITC protein was confirmed by observing growth inhibition and EPS production in *M. nivale* (Figure 4A). Fluorescent images of rmTAD1-FITC demonstrated that rmTAD1-FITC accumulated to a high level within the EPS layer (Figure 4B). Subsequent treatment with 2% cellulase decreased the levels of EPS around the hyphae (Figure 4C) and concomitantly diminished fluorescence signals (Figure 4D). These data suggested that the EPS exhibits some affinity to TAD1. This observation supported a putative function of EPS produced by *M. nivale* in the protection from the host plant defense system.

## 3. Discussion

**Figure 5:**
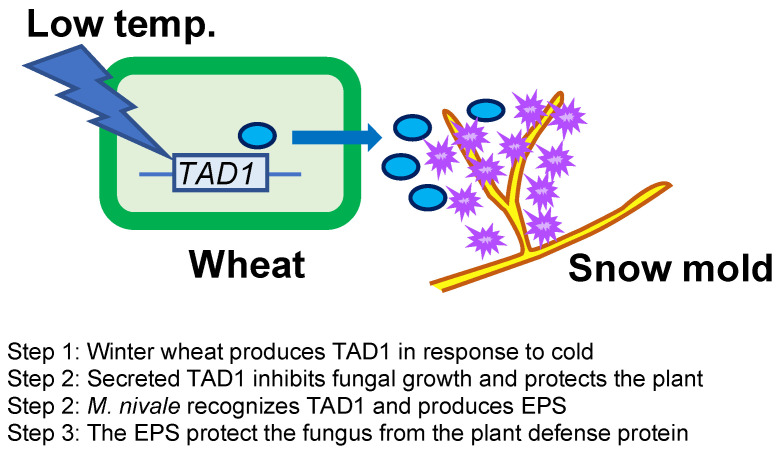
A proposed model for interaction of defense mechanisms between wheat and *M. nivale*.

Overwintering plants are known to acquire disease resistance during cold acclimation [2,4]. This mechanism is distinct from other induced defense mechanisms of plants in that it proceeds prior to infection and is considered as a preventative adaptation. Here, we demonstrated that TAD1 accumulates in the apoplast during cold acclimation (Figure 1 and Figure 2) and exhibits an antifungal activity against *M. nivale* (Figure 3A,B). It is known that apoplast is the extracellular compartment, which is a major battlefield of plants and pathogens [11]. Therefore, it is reasonable to consider that host plants secrete antimicrobial proteins to the apoplast as a defense response [12]. Our data suggested that TAD1 is involved in such a defense mechanism, although its accumulation is cold-induced and preventive (Figure 5).

We observed EPS production in *M. nivale* in response to TAD1 and detected colocalization of TAD1 with the EPS (Figure 3C and Figure 4). Collectively, these data suggest a function of the EPS as a protective barrier from antifungal proteins (Figure 5). A similar phenomenon was reported for *Magnaporthe grisea*, where polysaccharide α-1,3-glucan is induced by plant wax and protects the hyphal cell wall from digestive enzymes produced by plants [13]. In addition, it was also demonstrated that the activity of human antimicrobial peptides was suppressed in the presence of polysaccharides produced by lung pathogens [14]. We hypothesized that *M. nivale* produces EPS to protect its hyphae from plant antimicrobial proteins through direct interaction. It is not known, however, how *M. nivale* perceives the presence of antimicrobial proteins. In plants, pathogen-derived molecules, known as microbe-associated molecular patterns (MAMPs), are perceived as signals that triggers induction of immune responses [15]. In addition, endogenous molecules released from damaged cells after pathogen attack are recognized as danger signals [15,16]. It is reasonable to consider that similar mechanisms may be possible for the pathogens to recognize host defense mechanisms. Physiological changes caused by TAD1 may trigger a signal that induces the production of EPS. On the other hand, it is also possible that TAD1 can be recognized as a molecular pattern. Although plant–pathogen interactions have been extensively studied, very few studies have focused on the pathogen’s responses to plant defense systems. EPS production in response to TAD1 treatment may be utilized as a model system to decipher this novel plant–microbe interaction.

## 4. Conclusions

Wheat accumulates an apoplastic defensin, TAD1, during cold acclimation as a preventive response against snow molds. The pink snow mold, *M. nivale*, counteract the plant defense by producing EPS that capture TAD1.

## 5. Materials and Methods

### 5.1. Plant Growth and Cold Acclimation Conditions

Seeds of winter wheat (*Triticum aestivum* L. cv. Chihokukomugi) were sown in commercial potting mix and grown in a growth chamber that was maintained under 20 °C/15 °C (16 h light/8 h dark) cycles for 3 weeks. Cold acclimation was conducted by transferring plants to 2 °C (10 h light/14 h dark) in an environmentally controlled growth room. Plants were harvested prior to and during 14 d of cold treatment. The light intensity was set to 256 µmol m^−2^ s^−1^.

### 5.2. Extraction of Apoplastic Proteins

Apoplastic proteins were extracted as previously described by Hon et al. [17], with slight modifications. Leaf blades (50 g) of non-acclimated and cold-acclimated wheat plants were harvested and cut into 3 cm segments. These segments were gathered and tied, and the cut surface was washed with deionized water. Subsequently, the leaf segments were soaked in an extraction buffer (20 mM ascorbic acid, 20 mM CaCl_2_, pH 3.0) and vacuum infiltrated for 30 min. The leaf segments were then surface-dried with a paper towel and then placed in a plastic syringe barrel (10 mL). Afterwards, the syringe barrel was inserted into a 15 mL conical tube. Apoplastic fluids were recovered by centrifugation at 2000× *g* for 10 min using a swing out rotor. The fluid collected at the bottom of the tube was subsequently concentrated with a Microcon YM-3 (Millipore, Burlington, MA, USA). Protein concentration was estimated by using the Bio-Rad Protein Assay Kit (Bio-Rad, Hercules, CA, USA), and BSA was used as a standard.

### 5.3. Western Blot Analysis

Extracted apoplastic protein (4 µg) or soluble protein (25 µg) fractions were separated by SDS-PAGE and transferred onto Hybond-C extra membrane (GE Healthcare, Chicago, IL, USA). Hybridization was performed with primary polyclonal rabbit serum, which was raised against recombinant mature TAD1 (1:1000 *v*/*v*) and anti-rabbit IgG peroxidase-linked secondary antibody (1:10,000 *v*/*v*) (GE Healthcare, Chicago, IL, USA). Chemiluminescent detection of the signal was carried out with the ECL kit (GE Healthcare, Chicago, IL, USA) according to the manufacturer’s instructions.

### 5.4. Localization of GFP-Fused Protein

The entire coding sequence and the putative signal sequence (SigTAD1) of TAD1 was fused with a synthetic green fluorescent protein, sGFP (S65T) [18], and utilized as a reporter gene for TAD1 localization. As a reference, we used a marker gene for ER localization (DsRed-HDEL) [19,20]. Onion (*Allium cepa*) bulb scales were cut into small squares and placed onto a Murashige and Skoog (MS) agar plate (pH 8.4). TAD1-GFP, SigTAD1-GFP, and DsRed-HDEL plasmids were delivered into onion epidermal cells using particle bombardment. Gold particles (1.0 µm; Bio-Rad, Hercules, CA, USA) were coated with plasmid DNA according to the manufacturer’s instructions. Particles were bombarded into the onion epidermal cells using a Biolistic PDS-1000/He system (Bio-Rad, Hercules, CA, USA) with 1100 psi rupture discs that were operated under a vacuum condition. After bombardment, cells were allowed to recover for 18–22 h on the MS agar medium at 25 °C in the dark. Expression of the fusion constructs was monitored by using a Leica fluorescent microscopic system (FW4000). The filter sets used were the GFP filter (excitation; 470 nm, emission; 525 nm) for GFP fusion protein and the RFP filter (excitation; 546 nm, emission; 605 nm) for DsRed-HDEL protein, respectively.

### 5.5. Recombinant Protein Purification

Purification of recombinant mature TAD1 protein fused with GST (GST-mTAD1) was carried out as previously described [5]. GST-mTAD1 was induced by the treatment with IPTG (0.5 mM) for 4 h at 30 °C. Soluble proteins were recovered with the BugBuster^®^ Protein Extraction Reagent (Merck, Darmstadt, Deutschland). Affinity purification of the recombinant protein was performed by using a glutathione-sepharose 4B column (GE Healthcare, Chicago, IL, USA) and GST-mTAD1 was digested with PreScission^®^ Protease (GE Healthcare, Chicago, IL, USA) for 8 h at 4 °C according to the supplier’s instructions. The purified rmTAD1 was concentrated by a Microcon YM-3 column (Millipore, Burlington, MA, USA), and the concentration was determined with the Bio-Rad Protein Assay Kit (Bio-Rad, Hercules, CA, USA) using bovine serum albumin (BSA) as a standard.

### 5.6. Antifungal Activity of TAD1

Antifungal activity was analyzed as described previously, with slight modifications [21]. *M. nivale* (MCW222-7) [22] was cultured on potato dextrose agar (PDA; BD, Franklin Lakes, NJ, USA) medium at 4 °C for one month. Potato dextrose medium (20 mL) was inoculated with one agar plug (5 mm in a diameter) of *M. nivale* grown on PDA medium and cultured at 10 °C for 5 days with shaking at 120 rpm. The culture was crushed twice for 15 s with a polytron homogenizer (Kinematica AG, Littau, Switzerland). To test the hyphal growth inhibitory effect of TAD1, we cultured fragmented hyphae with 0, 2, 10, and 20 µg/mL rmTAD1 for approximately 9 days at 10 °C in a 96-well microtiter plate. Growth was measured by determining optical density at 595 nm in a spectrophotometer.

To test the inhibitory effect of TAD1 against spore germination, we cultured *M. nivale* on PDA medium at 10 °C for one month under a black light lamp. After one month, spores were collected with water and 20 µL of spore suspension (1 × 10^−6^ conidia mL^−1^) was mixed with 40 µL of PBS or rmTAD1 solution in PBS and 140 µL potato dextrose medium in a 96-well plate. After a 9 day incubation at 10 °C, the mixtures were stained with 10 µL of 1% Congo red solution, subsequently washed three times with distilled water, and observed through a differential interference contrast (DIC) microscope.

### 5.7. FITC Labeling

Labeling of rmTAD1 with FITC (Wako Pure Chemical industries, Osaka, Japan) was performed according to the manufacturer’s instructions.

## Data Availability

The data presented in this study are available in this article.

## Supplementary Materials

The following are available online at https://www.mdpi.com/article/10.3390/plants10081607/s1, Figure S1: Western blot analysis of cold-acclimated wheat total protein extracts.

## References

[1] Guy C.L. (1990). Cold acclimation and freezing stress tolerance: Role of protein metabolism. Annu. Rev. Plant Physiol. Plant Mol. Biol..

[2] Nakajima T., Abe J. (1996). Environmental factors affecting expression of resistance to pink snow mold caused by *Microdochium nivale* in winter wheat. Can. J. Bot..

[3] Tronsmo A.M., Gregersen P., Hjeljord L. (1993). Cold-Induced Disease Resistance. Mechanisms of Plant Defense Responses.

[4] Kuwabara C., Sasaki K., Umeki N., Hoshino T., Saburi W., Matsui H., Imai R. (2021). A model system for studying plant–microbe interactions under snow. Plant Physiol..

[5] Koike M., Okamoto T., Tsuda S., Imai R. (2002). A novel plant defensin-like gene of winter wheat is specifically induced during cold acclimation. Biochem. Biophys. Res. Commun..

[6] Ali S., Ganai B.A., Kamili A.N., Bhat A.A., Mir Z.A., Bhat J.A., Tyagi A., Islam S.T., Mushtaq M., Yadav P. (2018). Pathogenesis-related proteins and peptides as promising tools for engineering plants with multiple stress tolerance. Microbiol. Res..

[7] Sasaki K., Kuwabara C., Umeki N., Fujioka M., Saburi W., Matsui H., Abe F., Imai R. (2016). The cold-induced defensin TAD1 confers resistance against snow mold and Fusarium head blight in transgenic wheat. J. Biotechnol..

[8] Schweiger-Hufnagel U., Ono T., Izumi K., Hufnagel P., Morita N., Kaga H., Morita M., Hoshino T., Yumoto I., Matsumoto N. (2000). Identification of the extracellular polysaccharide produced by the snow mold fungus. Microdochium nivale. Biotechnol. Lett..

[9] François F., Lombard C., Guigner J.M., Soreau P., Brian-Jaisson F., Martino G., Vandervennet M., Garcia D., Molinier A.L., Pignol D. (2012). Isolation and characterization of environmental bacteria capable of extracellular biosorption of mercury. Appl. Environ. Microbiol..

[10] Aslam S.N., Newman M.A., Erbs G., Morrissey K.L., Chinchilla D., Boller T., Jensen T.T., De Castro C., Ierano T., Molinaro A. (2008). Bacterial Polysaccharides Suppress Induced Innate Immunity by Calcium Chelation. Curr. Biol..

[11] Doehlemann G., Hemetsberger C. (2013). Apoplastic immunity and its suppression by filamentous plant pathogens. New Phytol..

[12] Naz R., Bano A., Wilson N.L., Guest D., Roberts T.H. (2014). Pathogenesis-related protein expression in the apoplast of wheat leaves protected against leaf rust following application of plant extracts. Phytopathology.

[13] Fujikawa T., Kuga Y., Yano S., Yoshimi A., Tachiki T., Abe K., Nishimura M. (2009). Dynamics of cell wall components of *Magnaporthe grisea* during infectious structure development. Mol. Microbiol..

[14] Benincasa M., Mattiuzzo M., Herasimenka Y., Cescutti P., Rizzo R., Gennaro R. (2009). Activity of antimicrobial peptides in the presence of polysaccharides produced by pulmonary pathogens. J. Pept. Sci..

[15] Choi H.W., Klessig D.F. (2016). DAMPs, MAMPs, and NAMPs in plant innate immunity. BMC Plant Biol..

[16] Tanaka K., Choi J., Cao Y., Stacey G. (2014). Extracellular ATP acts as a damage-associated molecular pattern (DAMP) signal in plants. Front. Plant Sci..

[17] Hon W.-C., Griffith M., Chong P., Yang D.S.C. (1994). Extraction and isolation of antifreeze proteins from winter rye (*Secale cereale* L.) leaves. Plant Physiol..

[18] Niwa Y., Hirano T., Yoshimoto K., Shimizu M., Kobayashi H. (1999). Non-invasive quantitative detection and applications of non-toxic, S65T-type green fluorescent protein in living plants. Plant J..

[19] Okamoto T., Shimada T., Hara-Nishimura I., Nishimura M., Minamikawa T. (2003). C-terminal KDEL sequence of a KDEL-tailed cysteine proteinase (sulfhydryl-endopeptidase) is involved in formation of KDEL vesicle and in efficient vacuolar transport of sulfhydryl-endopeptidase. Plant Physiol..

[20] Nakaminami K., Karlson D.T., Imai R. (2006). Functional conservation of cold shock domains in bacteria and higher plants. Proc. Natl. Acad. Sci. USA.

[21] Spelbrink R.G., Dilmac N., Allen A., Smith T.J., Shah D.M., Hockerman G.H. (2004). Differential Antifungal and Calcium Channel-Blocking Activity among Structurally Related Plant Defensins. Plant Physiol..

[22] Christova P.K., Christov N.K., Imai R. (2006). A cold inducible multidomain cystatin from winter wheat inhibits growth of the snow mold fungus. Microdochium nivale. Planta.

