# Supplementary material for: An Apoplastic Defensin of Wheat Elicits the Production of Extracellular Polysaccharides in Snow Mold
Source: Plants (Basel). 2021 Aug 5;10(8):1607. doi: 10.3390/plants10081607 (PMC8400062; doi:10.3390/plants10081607)
Supplement: Supplementary file 1 [file plants-10-01607-s001.zip › plants-1323592-supplementary.pdf]

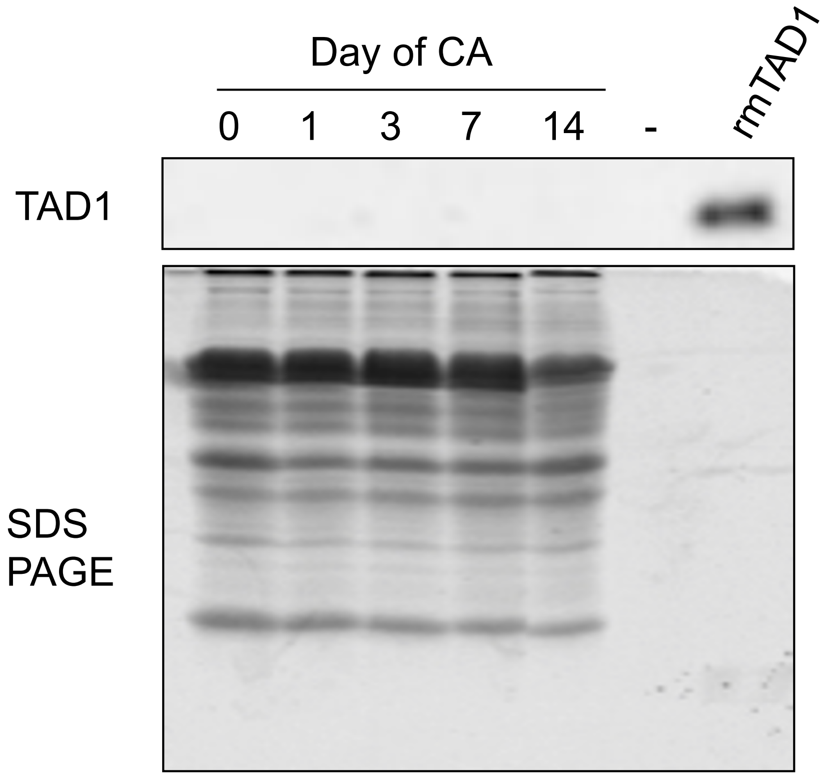

Figure S1. Western blot analysis of cold-acclimated wheat total protein extracts. Total proteins from a time-course of cold treatment were separated by SDS-PAGE and a duplicated gel was transferred to a membrane for Western blot analysis. Recombinant mature TAD1 (rmTAD1) was used as positive control for analysis. CA, cold acclimation.
